# Analysis of influencing factors on vibration characteristics of electro-hydraulic vibration cutting system

**DOI:** 10.1038/s41598-023-45329-3

**Published:** 2023-10-23

**Authors:** Miao Xie, Qingshuang Meng, Wenzhuo Zhang, He Wang, Jianfeng Guo, Yuan Liu, Wenxing Yan, Bo Li

**Affiliations:** 1 School of Mechanical Engineering, Liaoning University of Engineering and Technology, Fuxin, 123000 China; 2 Aerospace Heavy Engineering Equipment Co., Ltd, Wuhan, 430000 China; 3 School of Electrical and Control Engineering, Liaoning University of Engineering and Technology, Huludao, 125000 China

**Keywords:** Mechanical engineering, Coal

## Abstract

The cutting process of the cantilever tunneling machine mainly generates excitation through the cutting motor or the hydraulic cylinder driven by the hydraulic system. Regardless of the driving method, the frequency width of the excitation system is limited, difficult to control, and the excitation effect is poor. Therefore, in order to improve excavation efficiency, the excitation system parallel to the original cutting and rotating system is designed. Based on the excitation characteristics caused by alternating fluid flow, the core component of the excitation system, hydraulic exciter, is designed, and the dynamics and dynamic characteristics of the excitation system are analyzed. Based on AMESim software, analyze the impact of factors such as pump displacement, excitation frequency, and pipeline parameters on the operational performance of the electro-hydraulic vibration cutting system, and conduct experimental verification by building a cutting test bench. The experimental results show that as the inner diameter of the pipeline increases, the fluctuation at the acceleration turning point decreases in each cycle and approaches the peak faster. As the excitation frequency increases, the cutting acceleration of the electro-hydraulic excitation cutting system decreases first and then increases, verifying the accuracy of the simulation results. In the experiment, it was found that the acceleration transformation range of the cutting head of the excitation system is the smallest and most stable when the excitation frequency is 30 Hz. In order to verify that the excitation frequency of 30 Hz is the optimal frequency, further contact force tests were conducted on the cutting teeth. It was found that when the hydraulic excitation system adds a 30 Hz excitation frequency, the contact force on the cutting teeth is the smallest. It is more conducive to reducing the damage and wear of the cutting head, and the cutting effect of the cutting head is more obvious. The research results are beneficial for improving the cutting performance of the electro-hydraulic excitation cutting system, providing theoretical support for the selection of cutting parameters for excavation machinery and hydraulic excitation, and improving the existing theoretical system for active excitation cutting vibration analysis of excavation machines.

## Introduction

The rock breaking performance of the cutting system, as an important factor in promoting the reliability and operational efficiency of mining machinery, has been a focus of widespread attention by domestic and foreign researchers^1–3^. In response to the low cutting efficiency and high tooth loss of traditional rotary cutting methods, various vibration and impact assisted rock cutting methods have emerged. As the most commonly used excitation method, electro-hydraulic vibration has been widely studied by scholars at home and abroad.

Misra A^4^ et al. established a self-excited vibration system through a single cone valve, studied the correlation between valve flow coefficient and valve core displacement, and obtained the function relationship between valve core opening and valve load stiffness. The parameter optimization of the selfexcited vibration system still needs to be studied. Ruan^5^ et al. proposed an electro-hydraulic exciter scheme, designed a high-frequency 2D servo valve, and established a dynamic model of the servo exciter, which was verified through experiments. Wei^6^ et al. designed a new type of box type excitation valve and conducted numerical simulation analysis on the influence of valve input pressure, flow area ratio, and pre compression spring stiffness on the excitation characteristics of the system. Further theoretical verification is needed through experiments. Liu^7^ et al. proposed a multi shoulder valve core type rotary servo valve and excitation system, and studied the influence of valve core structure on the gain of the rotary valve. In addition, external factors should also be considered for their influence. Xiong Wenhui^8^ conducted in-depth research and feasibility analysis on several methods that can achieve hydraulic excitation based on a clear understanding of the excitation principle and feasible solutions of the tamping device. By comparing their excitation principle, structural form, reliability, feasibility, etc., the final design of the combination of servo valve and servo cylinder to achieve hydraulic excitation was selected. Wang Yuanchao^9^ conducted research on the speed of the valve core from two aspects: firstly, studying the influencing factors of the valve core speed and exploring its influencing laws; Secondly, using system identification to obtain the mathematical model of the proportional speed control valve controlled motor system, designing a PID controller to reduce the oscillation of the valve core speed and improve system stability. Long Xiaojian^10^ designed a rotary electro-hydraulic servo valve with a valve core, visualized the working process of the valve, and studied the static and dynamic working characteristics of the valve. The drawback is that there is a lack of analysis of the factors affecting the hydraulic power of the rotary valve, and the smoothness of the rotary valve operation needs further verification. Wang Tao^11^ developed a dual valve controlled electro-hydraulic excitation device based on the displacement combination of two unit valve cores, which can flexibly adjust the waveform frequency, amplitude, and vertical deviation in the excitation system, combining the existing rotary valve controlled electro-hydraulic excitation method. The adjustment characteristics of its output excitation waveform were studied. Liu Zhi^12^ et al. developed a two degree of freedom rotary valve with adjustable operating frequency. Based on AMESim software, the effects of parameters such as different commutation frequencies, oil supply pressure, and axial opening degree on the working characteristics of the valve were analyzed. The research object is a two degree of freedom rotary valve, which can be considered as the technology matures. Li Xiaopeng et al.^13^ designed a five port rotary valve based on the cyclic vibration law of continuous pile driving, analyzed the influence of rotary valve parameters on the characteristics of the pile driving system, and obtained the optimal rotary valve parameters for the pile driving system. The article only studied the effects of frequency and rotary valve aperture on pile driving efficiency, without considering other factors.

Starting from enhancing rock breaking ability and improving excavation efficiency, this article designs a vibration excitation system parallel to the original cutting and rotating system. Design the core component of the excitation system, hydraulic exciter, based on the excitation characteristics caused by alternating fluid flow, and construct a motion model for the flow process of the hydraulic exciter. Based on AMESim software, analyze the impact of factors such as pump displacement, excitation frequency, and pipeline parameters on the operational performance of the electro-hydraulic vibration cutting system, and conduct experimental verification by building a cutting test bench. The research results are beneficial for improving the theoretical system of active vibration cutting vibration analysis of existing tunneling machines, and can provide theoretical support for the selection of hydraulic vibration cutting parameters for tunneling machines.

## Vibration mechanism of electrohydraulic excitation system

**Figure 1:**
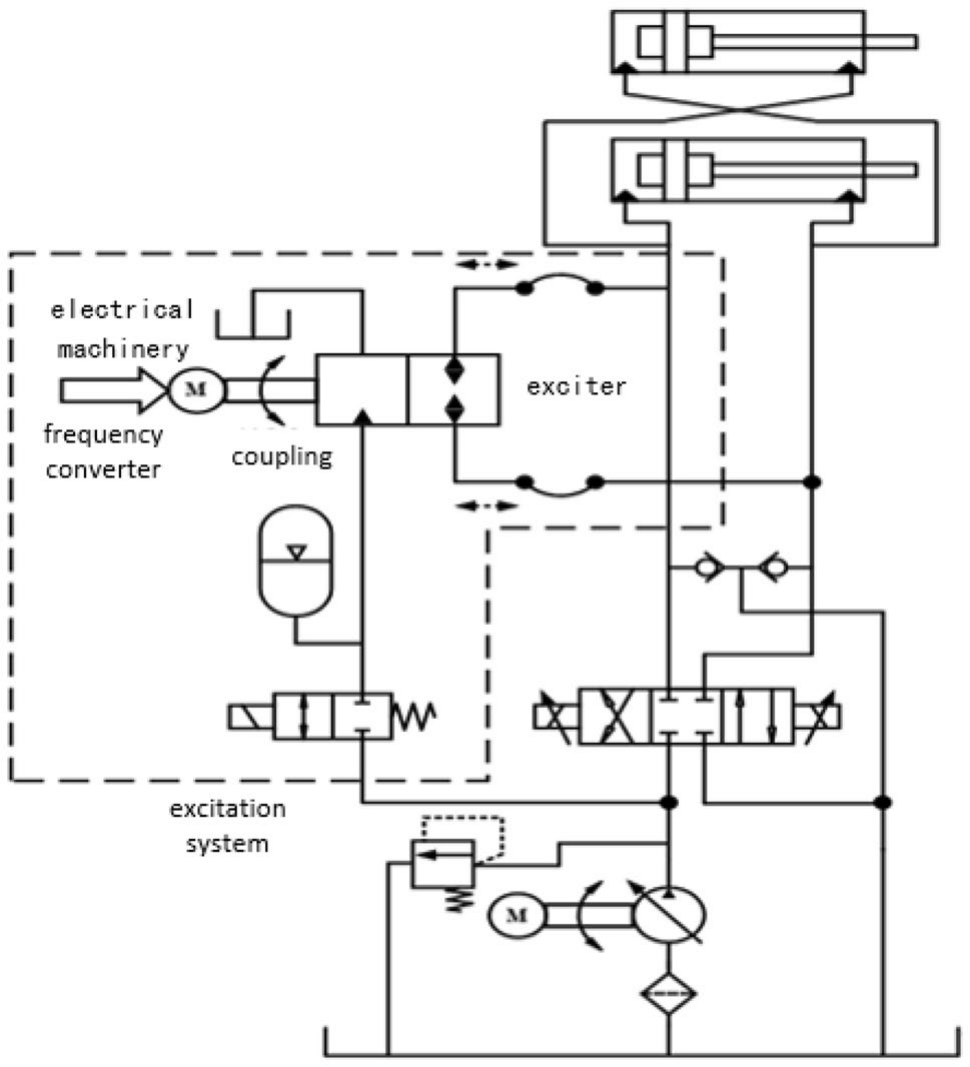
Schematic Diagram of Vibration Excitation System.

This article only analyzes the excitation system circuit, which excludes the original rotary cutting circuit. The hydraulic excitation system is composed of hydraulic excitation cylinders, hydraulic excitation devices (core components), frequency conversion mechanisms, hydraulic pipelines, and other components. The schematic diagram of the excitation system is shown in Fig. 1^14^. The principle of this excitation system is to control the hydraulic exciter through a frequency converter and an electric motor to maintain a fast switching state. The hydraulic excitation cylinder has a rod cavity and a rodless cavity to generate alternating liquid flow, and the piston rod undergoes reciprocating motion. The excitation cylinder is connected to the rotating end of the cutting part through hinge 1, and to the fixed end of the body through hinge 2, forming excitation. The design of the hydraulic excitation system is a hydraulic exciter (rotary valve) that generates alternating fluid flow.

According to the principle of active excitation, the optimal rock cutting performance is achieved when the system is in periodic motion and the response frequency is equal to the frequency of the excitation force. The excitation frequency of the excitation system should be close to the transverse response frequency of the cutting part's rotating condition, therefore, the excitation frequency is an important parameter of the excitation system^15,16^. The excitation force of the actuator must be greater than the resistance generated by the rotation of the cutting part during the vibration cutting process in order to produce a good excitation effect. The system amplitude, for coal rock and coal walls with different soil qualities, according to relevant data, the amplitude value at the cutting end should reach 5 mm in order to generate effective auxiliary cutting^17^. At the same time, when designing the hydraulic excitation system, it is necessary to consider: (1) The system frequency should not overlap with the natural frequency of key components to avoid resonance; (2) The output excitation force of the system must meet the resistance condition greater than that generated by the rotation of the cutting part; (3) The output amplitude of the system, i.e. the expansion and contraction of the hydraulic rod, should reach 2 mm at the corresponding frequency.

## Design and dynamic analysis of excitation system

This article addresses the shortcomings of traditional cutting methods that mainly rely on rotary cutting, and proposes a new auxiliary cutting method to enhance rock breaking ability and improve excavation efficiency. By improving the hydraulic system and generating active vibration characteristics to assist in cutting coal and rock. Build and solve the simulation model of the designed system scheme using AMESim software, analyze the pressure changes in the two chambers, and verify the accuracy of the built system simulation model. Analyze the variation patterns of excitation amplitude, velocity, and acceleration of the system output by changing the relevant variables of the system model (such as pump displacement, excitation frequency, pipeline parameters, etc.). Research on the dynamic performance of the system based on active excitation theory, with the goal of improving the cutting performance of the electro-hydraulic excitation cutting system, provides new ideas and methods for improving the cutting and rock breaking efficiency of tunneling machines. The following is an analysis of the structure, dynamics, and dynamic characteristics of the hydraulic excitation system.

**Figure 2:**
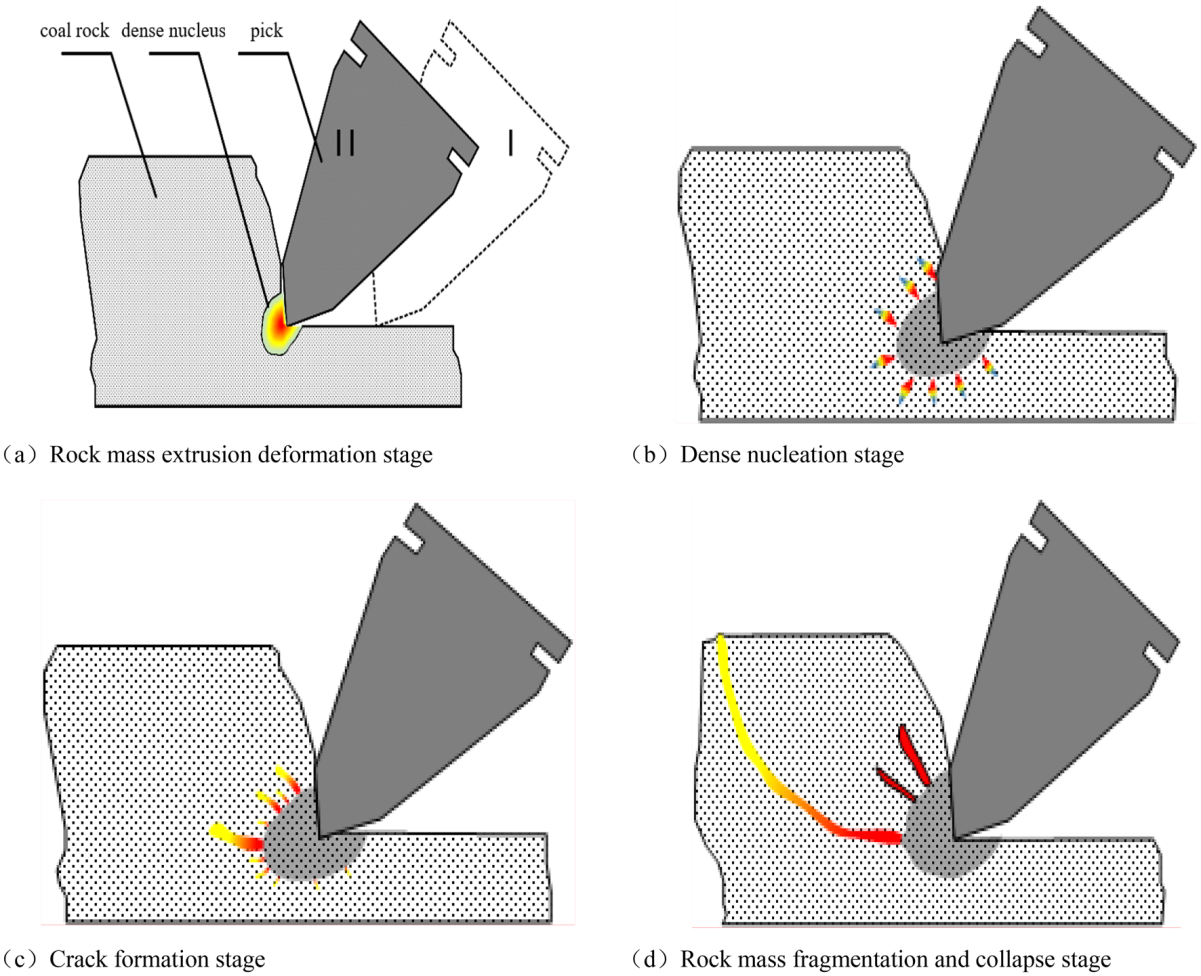
Rock breaking process of mechanical tools.

The following is a brief analysis of the rock breaking process of cutting tools using a pickaxe cutter as an example. The pickaxe cutter acts on coal and rock, and the tip of the cutter compresses the coal and rock mass to generate shear and tensile stress. As the stress increases and reaches the shear and tensile strength limit of the coal and rock mass, cracks appear inside the coal and rock mass, and the stress continues to rise. The cracks extend and the coal and rock mass fractures. The process of rock breaking under the action of a pickaxe cutter includes four stages: rock mass compression deformation, dense core formation, crack formation, and rock mass fragmentation and collapse, as shown in Fig. 2.

### Structural design of hydraulic vibration exciter

**Figure 3:**
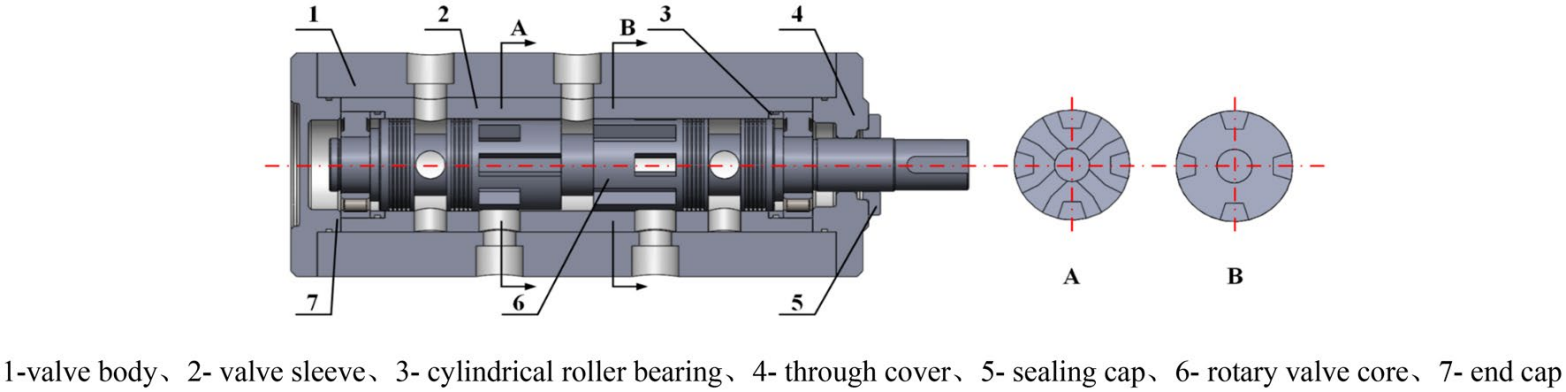
Schematic diagram of hydraulic vibrator structure and valve core section.

The hydraulic exciter, as the core component of the excitation system, is shown in Fig. 3. Its structural parameters affect the excitation frequency, flow rate, excitation amplitude, and other parameters of the excitation system.

The hydraulic exciter rotary valve core is machined with axial center holes, radial annular grooves, U-shaped grooves, and Y-shaped through-holes. When the valve core rotates, the U-shaped groove and Y-shaped through-hole alternate with the oil port, and there is a pressure equalizing groove machined at the shoulder to prevent radial force imbalance and avoid clamping conditions. The rotation of the valve core relies on cylindrical roller bearings, which have a low friction coefficient and are suitable for high-speed working conditions. The inner and outer rings are easy to separate, making it easy to disassemble and install. As a wearable accessory, the valve sleeve remains fixed to the valve body. Connect the motor and rotary valve using a coupling, and the motor drives the rotary valve for flow distribution. Above the rotary valve shown in the figure are the working oil ports B and A, which are connected to the rod and no rod chambers of the hydraulic vibration cylinder. The left side below is the system inlet port P, and the right side is the system return port T.

**Figure 4:**
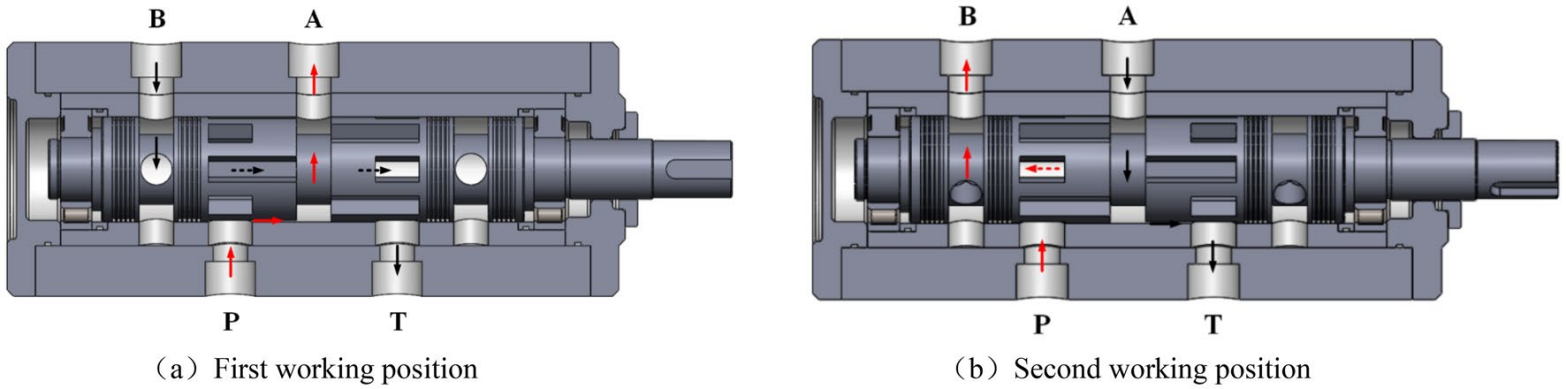
Working position of primary rotary valve spool excited by exciting cylinder.

On one side of the shoulder of the hydraulic rotary valve spool, there are alternating Y-shaped through-holes and U-shaped grooves, consisting of four groups. The spool completes one revolution and the vibration cylinder excites four times. As shown in Fig. 3, the schematic diagram of the first and second working positions of the valve core of the excitation cylinder and the flow direction of the working medium at the P, T, A, and B oil ports is shown in Fig. 4.

1. The first working position during the work process.
  The U-shaped groove of the non through-hole section is opposite to the P-port, and the Y-shaped through-hole is opposite to the T-port.
  Working medium → Oil inlet P → U-shaped groove → Annular groove → Port A → Cylinder rodless chamber.
  Working medium with rod cavity → Port B → Annular groove → Axial center hole → Y-shaped through-hole → Port T → Oil tank.
2. Second working position during the working process.
  The Y-shaped through-hole is opposite to the P-port, and the U-shaped groove on the non through-hole section is opposite to the T-port.
  Working medium → Port P → Y-shaped through-hole → Axial center hole → Annular groove → Port B → Rod chamber of oil cylinder.
  Rodless cavity working medium → Port A → Circular groove → U-shaped groove → Port T → Oil tank.

### Mathematical model of vibration cylinder motion

**Figure 5:**
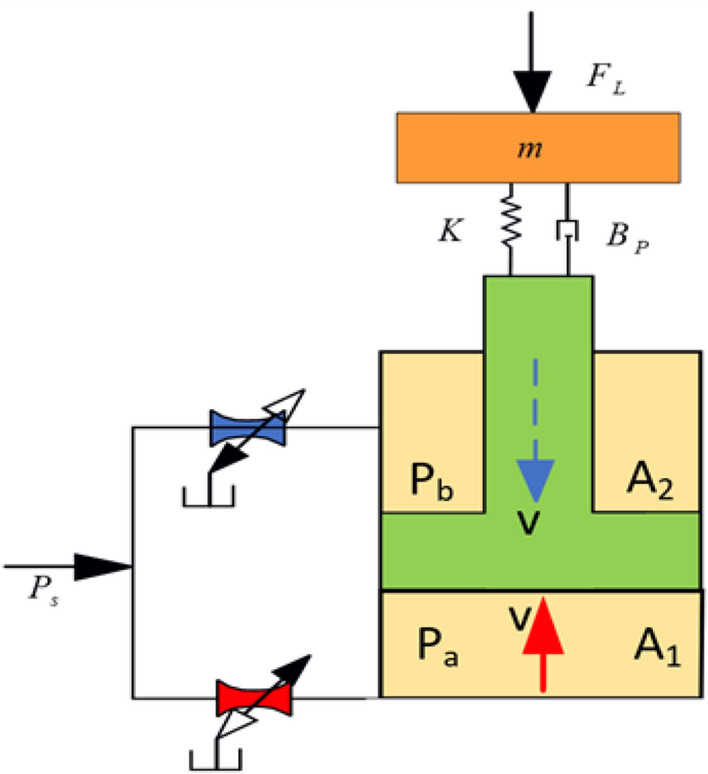
Motion model of exciting hydraulic cylinder.

As shown in Fig. 5, when the rod of the excitation cylinder moves upwards, the working medium enters the rodless chamber from the T and B ports of the rotary valve and the rodless chamber pipeline. At the same time, the working medium of the rodless chamber is returned to the hydraulic oil tank through the rodless chamber pipeline, rotary valve A, and P ports, and the piston moves up due to the pressure of the two chambers. Set the flow rate of rodless cavity—Q_1_ (t), flow rate of rodless cavity—Q_2_ (t), flow rate from port T to port B—Q_TB_, flow rate from port A to port P—Q_AP_, hydraulic system supply pressure—P_s_, rodless cavity pressure—P_1_, rodless cavity pressure—P_2_, rodless cavity connection pipeline pressure—P_I_, rodless cavity connection pipeline pressure—P_II_, and system pressure—P_b_. Area of rodless cavity—A_1_, area of rodless cavity—A_2_, area of rodless cavity connected pipeline—A_I_, area of rodless cavity connected pipeline—A_II_, and area of port—b. The volume of the vibration cylinder without rod cavity—V_1_, with rod cavity volume—V_2_, without rod cavity connecting pipeline volume—V_I_, with rod cavity connecting pipeline volume—V_II_, and the volume elastic modulus of the working medium- β_e_. The internal and external leakage coefficients C_ip_ and C_ep_ of the excitation cylinder.

That is, it can be obtained that the flow rate Q_2_ (t) of the rod cavity is:

1 $$ Q_{2} \left( t \right) = A_{2} \frac{dy}{{dt}} + \frac{{V_{2} + A_{2} y}}{{\beta_{e} }} \cdot \frac{{dP_{2} }}{dt} - C_{ip} \left( {P_{2} - P_{1} } \right) - C_{ep} P_{2} $$

2 $$ Q_{2} \left( t \right) = C_{d} a_{II} \sqrt {2\left( {P_{2} - P_{II} } \right)/\rho } .$$

The flow rate Q_1_ (t) of the rodless cavity is:

3 $$ Q_{1} \left( t \right) = A_{1} \frac{dy}{{dt}} + \frac{{V_{1} + A_{1} y}}{{\beta_{e} }} \cdot \frac{{dP_{1} }}{dt} - C_{ip} \left( {P_{2} - P_{1} } \right) - C_{ep} P_{1} $$

4 $$ Q_{1} \left( t \right) = C_{d} a_{I} \sqrt {2\left( {P_{I} - P_{1} } \right)/\rho }. $$

The flow rate from port T to port B—Q_TB_ is:

5 $$ Q_{TB} = C_{d} b\sqrt {2\left( {P_{II} - P_{s} } \right)/\rho } $$

6 $$ Q_{TB} = Q_{2} \left( t \right) + \frac{{V_{II} }}{{\beta_{e} }} \cdot \frac{{dP_{II} }}{dt} .$$

The flow rate from port A to port P—Q_AP_ is:

7 $$ Q_{AP} = C_{d} b\sqrt {2\left( {P_{I} - P_{b} } \right)/\rho } $$

8 $$ Q_{AP} = Q_{1} \left( t \right) - \frac{{V_{I} }}{{\beta_{e} }} \cdot \frac{{dP_{I} }}{dt}. $$

The force balance equation is constructed as follows:

9 $$ m\frac{{d^{2} y}}{{dt^{2} }} + c\frac{dy}{{dt}} + ky = A_{2} P_{2} - A_{1} P_{1} - mg. $$

By combining the above equation, a dynamic mathematical model of the upward movement of the piston rod in the hydraulic excitation system can be obtained. Assuming the state variable $x = \left[ {y,y^{\prime},P_{I} ,P_{1} ,P_{2} ,P_{II} } \right]^{T}$, the state space equation for the upward movement of the excitation system piston rod is as follows:

10 $$ \left\{ \begin{gathered} \dot{x}_{1} = x_{2} \hfill \\ \dot{x}_{2} = \frac{{A_{2} }}{m}x_{5} - \frac{{A_{1} }}{m}x_{4} - g - \frac{c}{m}x_{2} - \frac{k}{m}x_{1} \hfill \\ \dot{x}_{3} = \frac{{\beta_{e} }}{{V_{I} }}\left( {C_{d} a_{1} \sqrt {2\left( {x_{3} - x_{4} } \right)/\rho } - C_{d} b\sqrt {2\left( {x_{3} - P_{b} } \right)/\rho } } \right) \hfill \\ \dot{x}_{4} = \frac{{\beta_{e} }}{{V_{1} + A_{1} x_{1} }}\left( {A_{1} x_{2} - C_{d} a_{II} \sqrt {2\left( {x_{3} - x_{4} } \right)/\rho } - C_{ip} \left( {x_{5} - x_{4} } \right) - C_{ep} x_{5} } \right) \hfill \\ \dot{x}_{5} = \frac{{\beta_{e} }}{{V_{2} + A_{2} x_{1} }}\left( {A_{2} x_{2} - C_{d} a_{I} \sqrt {2\left( {x_{6} - x_{5} } \right)/\rho } - C_{ip} \left( {x_{5} - x_{4} } \right) - C_{ep} x_{4} } \right) \hfill \\ \dot{x}_{6} = \frac{{\beta_{e} }}{{V_{II} }}\left( {C_{d} b\sqrt {2\left( {x_{6} - P_{s} } \right)/\rho } - C_{d} a_{II} \sqrt {2\left( {x_{5} - P_{6} } \right)/\rho } } \right). \hfill \\ \end{gathered} \right. $$

### Dynamic analysis of excitation system

**Figure 6:**
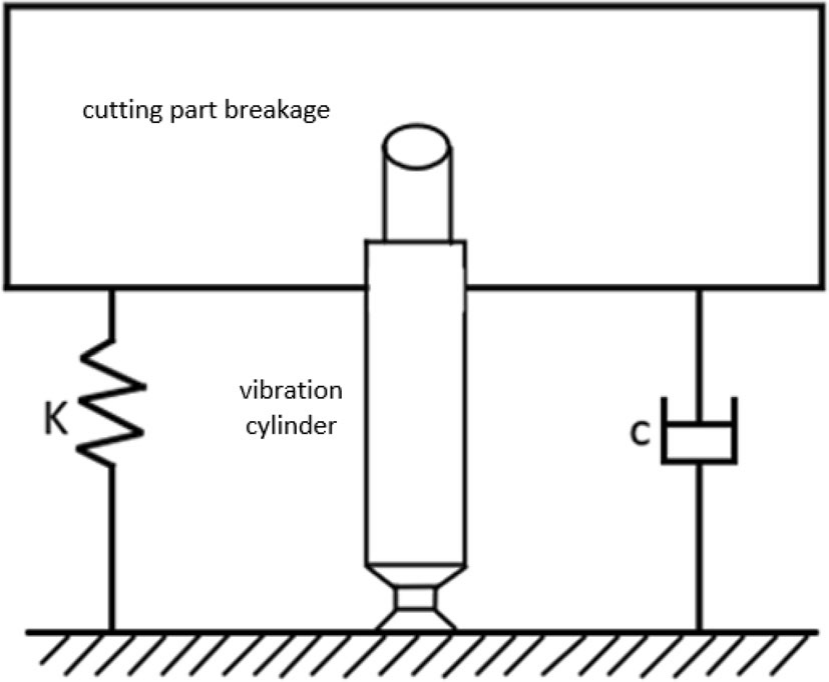
Mechanical Model of Hydraulic Excitation System.

Based on existing research^18,19^, the excitation cylinder serves as the actuator of the excitation system. The excitation cylinder is an executive component of the excitation system. Considering the alternating fluid flow effect of the excitation, a double acting piston type cylinder is selected. The size parameters are related to the mass of the cutting end, system damping, and stiffness. The mechanical model of the hydraulic excitation system is shown in Fig. 6.

Assuming that the oil is incompressible, the excitation force *F(t)* is decomposed into an invariant component *F*_*0*_ and a periodic component *f(t)*. The invariant component *F*_*0*_ only causes static displacement of the piston, and only the periodic component *f(t)* causes forced vibration of the piston. Therefore, the vibration equation of the system can be obtained as follows:

11 $$ f\left( t \right) = m\ddot{x} + c\dot{x} + kx, $$

where: *m* is the sum of effective mass of cutting part end and vibration cylinder moving parts, kg; *c* is the mechanical damping, air damping, etc. in the excitation system, N · s/m; *k* is the stiffness of the excitation system, N/m.

Expand the periodic component *f(t)* into Fourier series:

12 $$ f\left( t \right) = \frac{4F}{\pi }\sum\limits_{n = 1}^{\infty } {\frac{1}{2n - 1}} \sin \left( {2n - 1} \right)\omega t = \sum\limits_{{\begin{array}{*{20}c} {i = 2n - 1} \\ {n = 1} \\ \end{array} }}^{\infty } {F_{i} \sin i\omega t}, $$

where: *F*_*i*_ is the i-th order component amplitude of the excitation force, N; *ω* is the angular frequency of the excitation force, HZ.

By solving Eq. (11), the following equation can be obtained:

13 $$ x\left( t \right) = \sum\limits_{{\begin{array}{*{20}c} {i = 2n - 1} \\ {n = 1} \\ \end{array} }}^{\infty } {F_{i} \beta_{i} \sin \left( {{\text{i}}\omega {\text{t}} - \theta_{i} } \right)} = \sum\limits_{{\begin{array}{*{20}c} {i = 2n - 1} \\ {n = 1} \\ \end{array} }}^{\infty } {X_{i} \sin \left( {{\text{i}}\omega {\text{t}} - \theta_{i} } \right)} , $$

where: *X*_*i*_ is the i-th order displacement amplitude of the excitation displacement, m; *β*_*i*_ is the amplification factor of the i-th order fractional displacement of the excitation displacement; *θ*_*i*_ the phase angle of the i-th order fractional displacement of the i-excitation displacement, °.

14 $$ \beta_{i} = \frac{1}{K}\frac{1}{{\sqrt {\left[ {1 - \left( {i\omega /\omega_{0} } \right)^{2} } \right]^{2} } + \left( {2\xi i\omega /\omega_{0} } \right)^{2} }} = \frac{1}{K}\frac{1}{{\sqrt {\left[ {1 - \left( {ir} \right)^{2} } \right]^{2} } + \left( {2\xi ir} \right)^{2} }} $$

15 $$ \tan \theta_{i} = \frac{{2\xi i\omega /\omega_{0} }}{{1 - \left( {i\omega /\omega_{0} } \right)^{2} }} = \frac{2\xi ir}{{1 - i^{2} r^{2} }}, $$

where: *ω*_*0*_ is the natural frequency of the excitation system, $\omega_{0} = \sqrt {K/m}$, Hz;*ξ* is the damping ratio of the excitation system, $\xi = C/\left( {2\sqrt {mK} } \right)$; *r* is the ratio of excitation frequency to natural frequency, $r = \omega /\omega_{0}$.

When r ≥ 1, the displacement of the hydraulic excitation piston is

16 $$ X\left( t \right) = X_{1} \sin \left( {\omega t - \theta_{1} } \right). $$

The displacement amplitude of the piston is

17 $$ X_{1} = \frac{4F}{{\pi K}} = \frac{1}{{\sqrt {\left( {1 - r^{2} } \right)^{2} + \left( {2\xi r} \right)^{2} } }} $$

18 $$ X_{1} \le \frac{{Q_{1} }}{{\omega A_{1} }}, $$

where: *F* is the amplitude of the excitation force,mm; *A*_*1*_ is the area of the hydraulic rod, mm; *Q*_*1*_ is the flow rate of the exciter, L/min.

After determining the vibration parameters required by the system to excite the hydraulic cylinder, the flow rate is based on the maximum instantaneous flow rate of the system. The flow rate inside the pipeline is calculated at 5 m/s, and the inner diameter of the system pipeline is determined based on the formula $d = \sqrt {4Q/\pi \left[ v \right]}$. Therefore, the pipeline connecting the piston chamber of the excitation hydraulic cylinder is equal to $\sqrt 2$ the inner diameter of the pipeline connecting the rod chamber. Therefore, the relationship between pipeline area and hydraulic rod area is A = 1/2A_1_.

The relationship between the theoretical displacement and theoretical flow rate of a hydraulic pump is as follows:

19 $$ Q_{1} = Q \cdot n, $$

where: *Q* is the pump displacement, L/min; *n* is the pump speed, r/min.

Therefore, the following equation can be obtained:

20 $$ X_{1} \le \frac{2Qn}{{\omega \pi d^{2} }},F = \frac{nKQ}{{2d^{2} \omega }}. $$

From the above equation, it can be seen that the displacement amplitude of the excitation piston mainly depends on three factors: the excitation frequency of the exciter *ω*, Displacement *Q*, pipeline inner diameter *d*. Under certain system parameters, the amplitude of the excitation system can be changed by adjusting the system displacement, excitation frequency, and pipeline inner diameter. When other variables are constant, the amplitude of the excitation system increases with the increase of displacement. However, the excitation frequency and pipeline inner diameter are inversely proportional to the amplitude of the excitation system.

### Analysis of dynamic characteristics of excitation system

**Figure 7:**
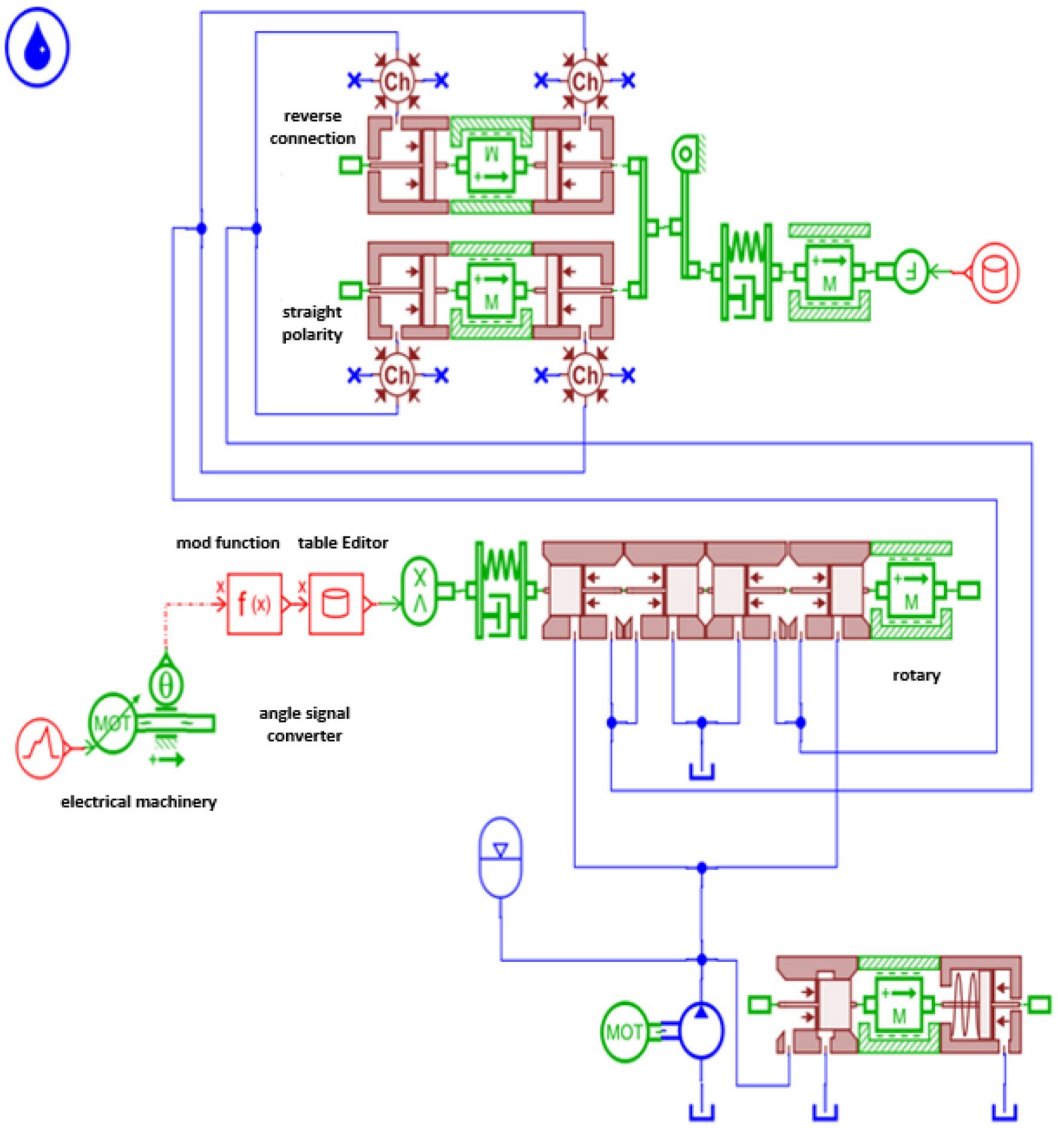
Simulation model of excitation system.

Based on the working principle of the excitation system, the system simulation model was built using AMESim software, as shown in Fig. 7.

**Figure 8:**
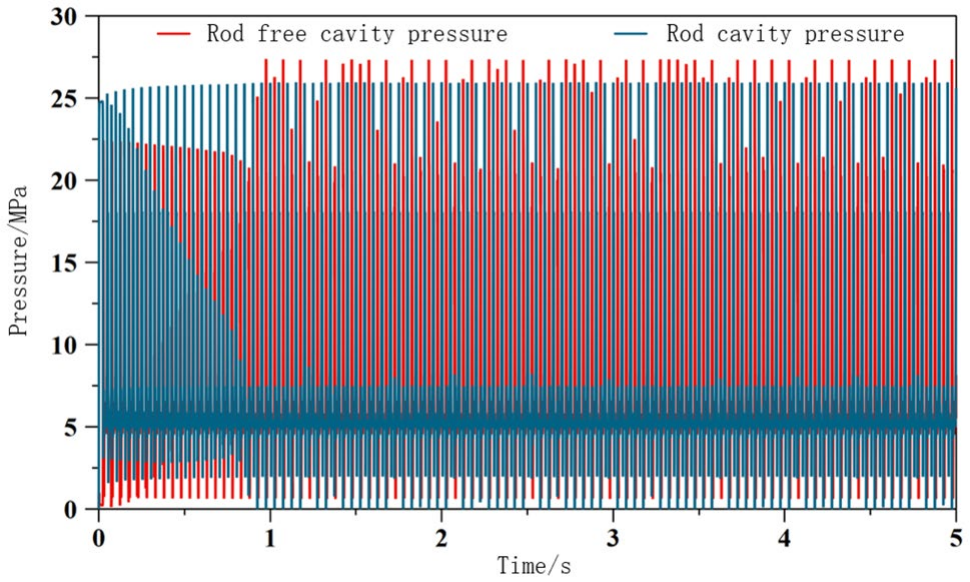
Pressure change of two chambers of excitation cylinder.

**Figure 9:**
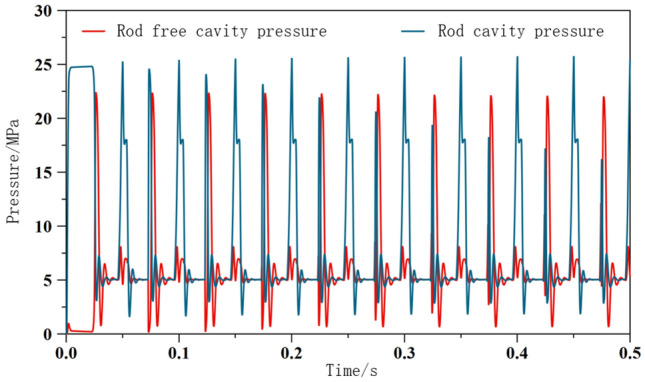
Local enlargement.

After constructing the excitation system model, set the parameters of each relevant component based on specific parameters, as shown in Table 1. And solve the system model, and demonstrate the excitation effect generated by the system by observing the pressure changes in the front and rear chambers of the excitation cylinder, as shown in Figs. 8 and 9. Study the dynamic performance (amplitude, velocity, acceleration) of the excitation system through changes in factors such as pump displacement, system excitation frequency, and pipeline parameters. Taking the direct system in the excitation system simulation model as an example, explore the impact of specific factors on the excitation system.

**Table 1:** Parameter settings.

Element	Parameter	Numerical value
Hydraulic pump	Hydraulic pump displacement/ml.r^−1^	60
Relief valve	system pressure upper limit/MPa	31.5
Signal source	Valve core motion frequency/Hz	30
Hydraulic exciter	Spool valve core diameter/mm	50
	Spool valve piston diameter/mm	25
Hydraulic cylinder	piston diameter/mm	50
	Piston rod diameter/mm	25
Motor	motor speed/r.min^−1^	1420
Vibration exciter and oil cylinder connection pipeline	Connecting pipe diameter/mm	25
	Connecting pipeline length/mm	800

Analyze the pressure in the front and rear chambers of the excitation cylinder, and explore the pressure changes by drawing a graph of the pressure changes in the front and rear chambers. As shown in Fig. 8, the pressure in each group with a rod chamber is slightly higher than that in the piston chamber, with a peak pressure of 27.33 MPa in the absence of a rod chamber and 25.94 MPa in the presence of a rod chamber. It can be seen that the pressure in the front and rear chambers of the excitation cylinder increases alternately, remains basically stable after 1 s, and cycles within a certain period, that is, the piston of the excitation cylinder undergoes reciprocating displacement movement under the periodic action of no rod chamber or rod chamber, generating excitation. At the same time, the frequency of pressure change is the same as the system setting frequency, which can prove that the system output frequency meets expectations and is basically consistent.

#### Analysis of the impact of pump displacement on the cutting system

**Figure 10:**
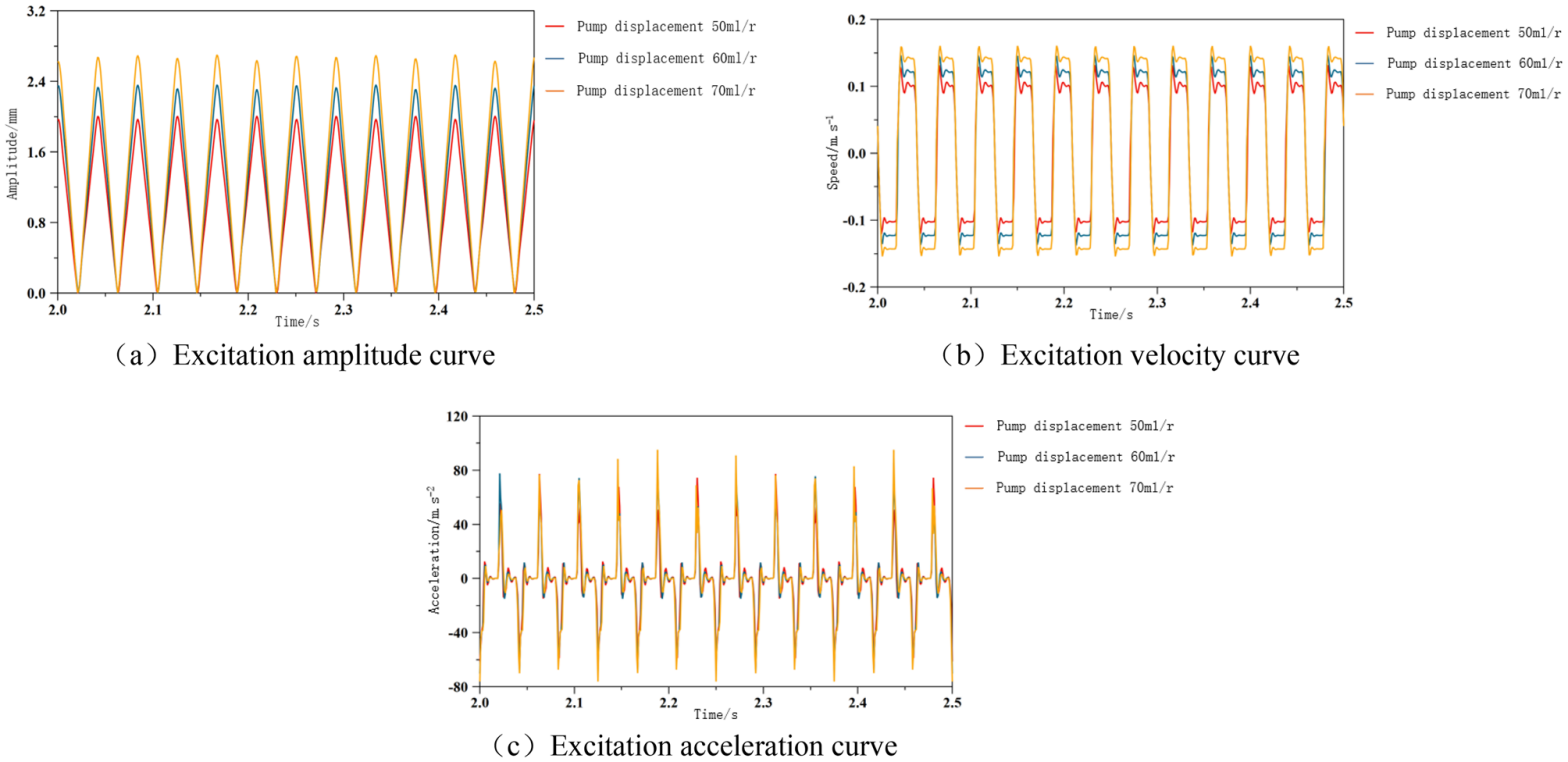
Excitation amplitude and acceleration curve under different pump displacement conditions.

As the power component of the hydraulic excitation system, the hydraulic pump is the power source for stable and normal operation of the system. The pump displacement only ignores the volume of working medium that can be discharged by rotating the pump spindle for one cycle in the case of internal and external leakage of the pump. According to formula (20), the relationship between the amplitude of the excitation system and the pump displacement can be concluded that changes in pump displacement have a certain impact on the dynamic performance characteristics of the hydraulic system. By ensuring that the excitation frequency and other parameters of the system remain unchanged, the pump displacement was adjusted to 50 ml/r, 60 ml/r, and 70 ml/r, respectively, to study the impact of pump displacement on the dynamic performance of the excitation system. The simulated curve is shown in Fig. 10.

From the simulation results in Fig. 10, it can be seen that under the condition of keeping other parameters constant, the excitation amplitude and speed of the pump displacement are in a positive proportion relationship. As the pump displacement increases, the amplitude and speed of the cutting system gradually increase, which is consistent with theory. The amplitude is adjusted by adjusting the pump displacement, but the excitation acceleration does not change with the displacement, and the peak values are maintained at ± 80 m/s^2^. Simultaneously changing the pump displacement has no effect on the excitation frequency period.

#### Analysis of the influence of excitation frequency on cutting system

**Figure 11:**
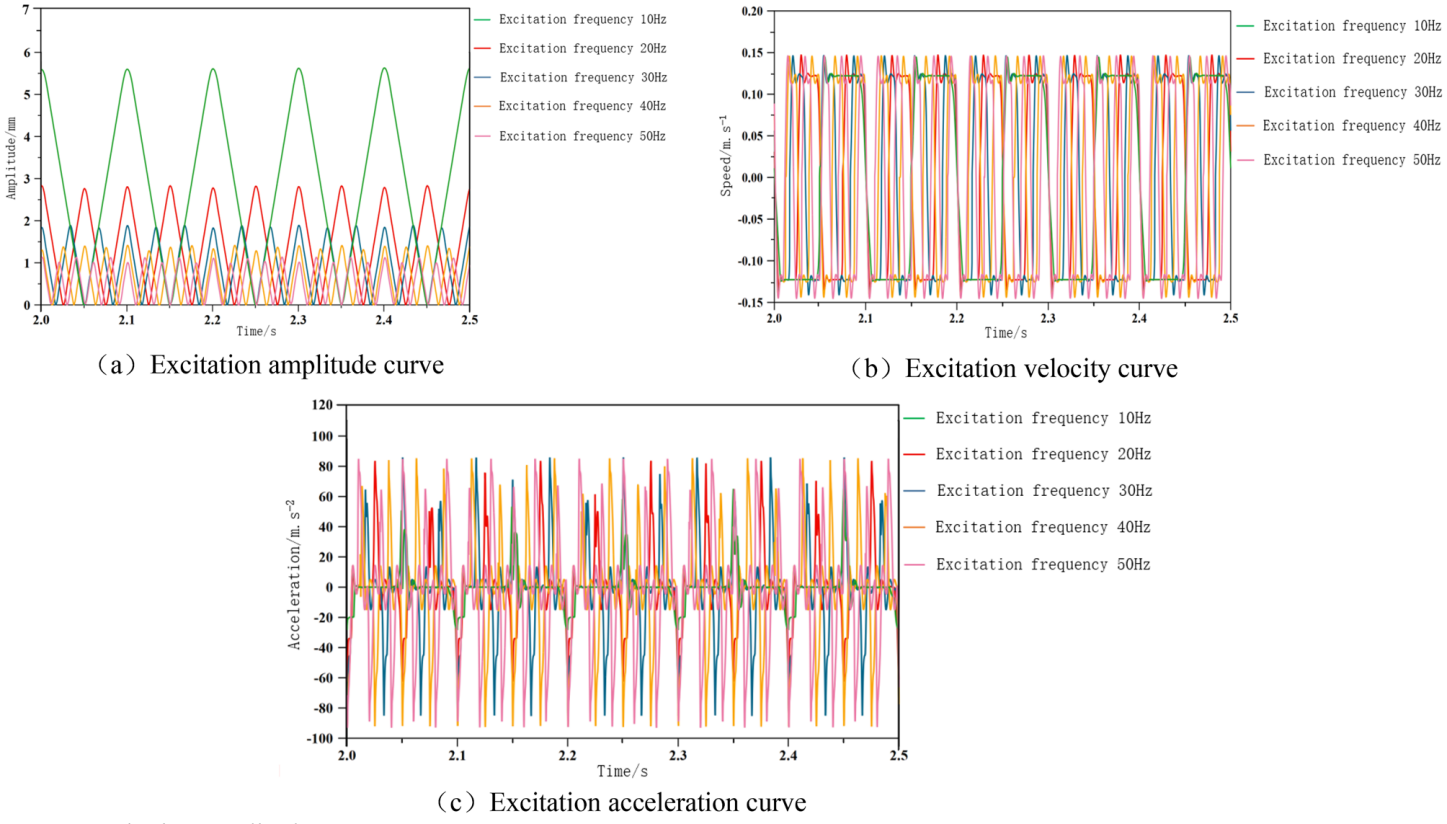
Excitation amplitude curve under different frequency conditions.

The hydraulic excitation rotary valve operates by rotating the valve core, and the flow area of the valve port periodically changes. The frequency of periodic changes directly affects the number of connections between the valve port and the working mechanism oil port per unit time, i.e. the excitation frequency. This article simulates the rotating state of the rotary valve through angle displacement sensor, mod residual function, and table editor. Frequency is a quantity that characterizes the degree of periodic change, defined as the number of times a periodic change is completed per unit time, in units of s^-1^. If the speed of the excitation motor is set to n, the system excitation frequency is (n/60) * the number of fluid flow changes when the valve core rotates one axis. By changing the motor speed, the valve core frequency can be controlled. According to formula (20), the relationship between the amplitude and frequency of the excitation system can be concluded that the change in excitation frequency has a certain impact on the dynamic performance characteristics of the hydraulic system. The frequency adjustment range of the hydraulic exciter used is 0–60 Hz. This article conducts simulation research using AMESim software and selects hydraulic excitation frequencies of 10 Hz, 20 Hz, 30 Hz, 40 Hz, and 50 Hz for analysis. The other parameters of the simulation control system remain unchanged each time, and the excitation frequency starts at 10 Hz, increases by 10 Hz each time, and continues until 50 Hz. The impact of several sets of excitation frequencies on the dynamic performance of the electro-hydraulic excitation cutting system is shown in Fig. 11.

According to the curve in Fig. 11, the change in excitation frequency has a significant impact on the dynamic performance of the excitation system. The higher the excitation frequency, the smaller the excitation amplitude of the excitation system, and the shorter the excitation period of the system, which means the rate of reaching the peak amplitude is accelerated. As the excitation frequency increases, the speed of the cutting system gradually increases, and the acceleration shows a trend of first decreasing and then increasing. The acceleration is the smallest when the excitation frequency is 30 Hz.

#### Analysis of the influence of hydraulic pipeline parameters on the cutting system

The hydraulic excitation system mainly connects power, execution, control, components, etc. through pipelines. The parameters of the working medium circulating in the pipeline have an impact on the dynamic performance characteristics of the system, such as whether the working medium between each circuit in the system can flow stably, that is, the transformation of hydraulic energy to other forms of energy, so that all parts of the system can work normally as expected.

**Figure 12:**
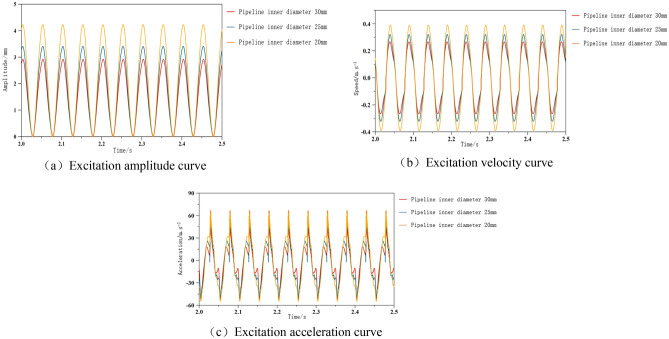
Performance curve of excitation system under different pipe diameters.

According to formula (20), the relationship between the amplitude of the excitation system and the inner diameter of the pipeline can be concluded that changes in the inner diameter of the pipeline have a certain impact on the dynamic performance characteristics of the hydraulic system. Adjust the inner diameter of the pipeline to 20 mm, 25 mm, and 30 mm, and analyze the dynamic performance of the excitation system for different inner diameters of the connected pipeline. By utilizing the batch processing function in AMESim, the three inner diameter sizes were set and solved to obtain the curves as shown in Fig. 12.

The systematic characteristics of excitation when the inner diameter of the pipeline is different are shown in Fig. 12. As shown in the figure, as the inner diameter of the pipeline increases, the peak excitation amplitude shows a decreasing trend, and the curve trend is the same. In terms of speed, as the inner diameter of the pipeline increases, the peak speed shows a decreasing trend, and the curve trend is the same. In terms of acceleration, as the inner diameter of the pipeline increases, the fluctuation at the acceleration turning point decreases in each cycle and approaches the peak faster. In addition, if the amplitude is adjusted through the pipeline, it is also necessary to consider that when the inner diameter of the pipeline is small, the oil temperature will rapidly rise under frequent alternating effects, resulting in phenomena such as cavitation, cavitation, and noise. From this, it can be seen that the internal pressure of the pipeline has a certain degree of impact on the excitation performance of the excitation process.

The influence trend of pump displacement, excitation frequency, and pipeline inner diameter on the cutting system was analyzed using simulation software. The conclusions obtained verify the accuracy of the mechanical model of the hydraulic excitation system in Sect. 3.4.

## Experimentation

**Figure 13:**
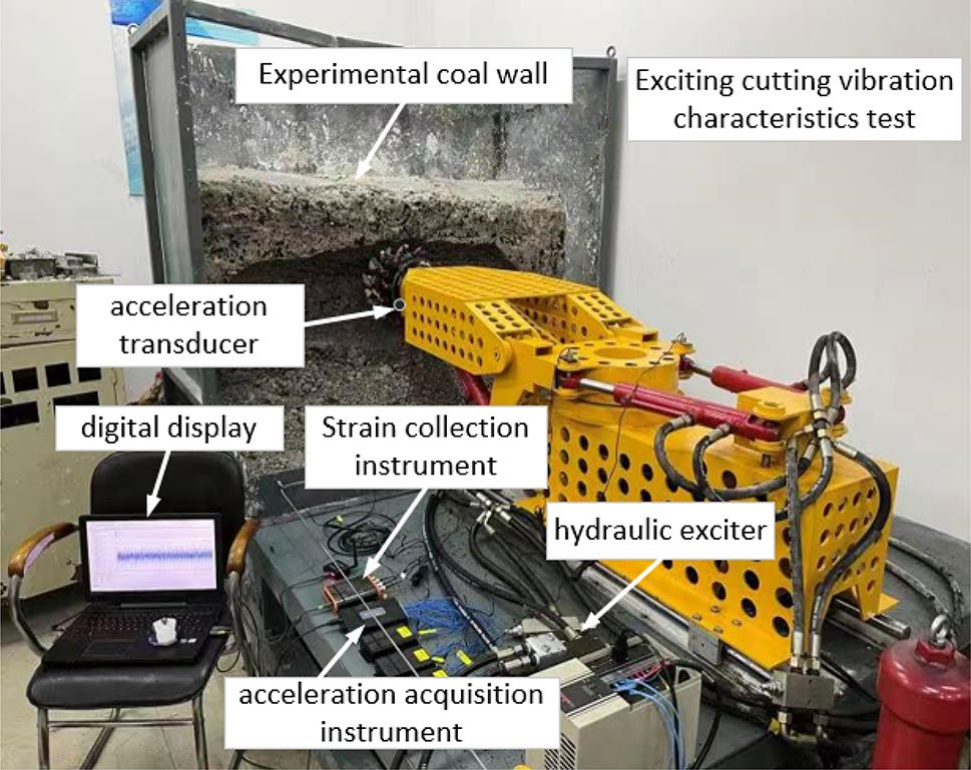
Cutting system test bench.

Using a self-made coal rock cutting experimental device^20^, the experimental coal wall is configured in a ratio of coal powder: cement: water = 1.62:1:0.49. The structure diagram of the cutting experimental platform is shown in Fig. 13.

**Figure 14:**
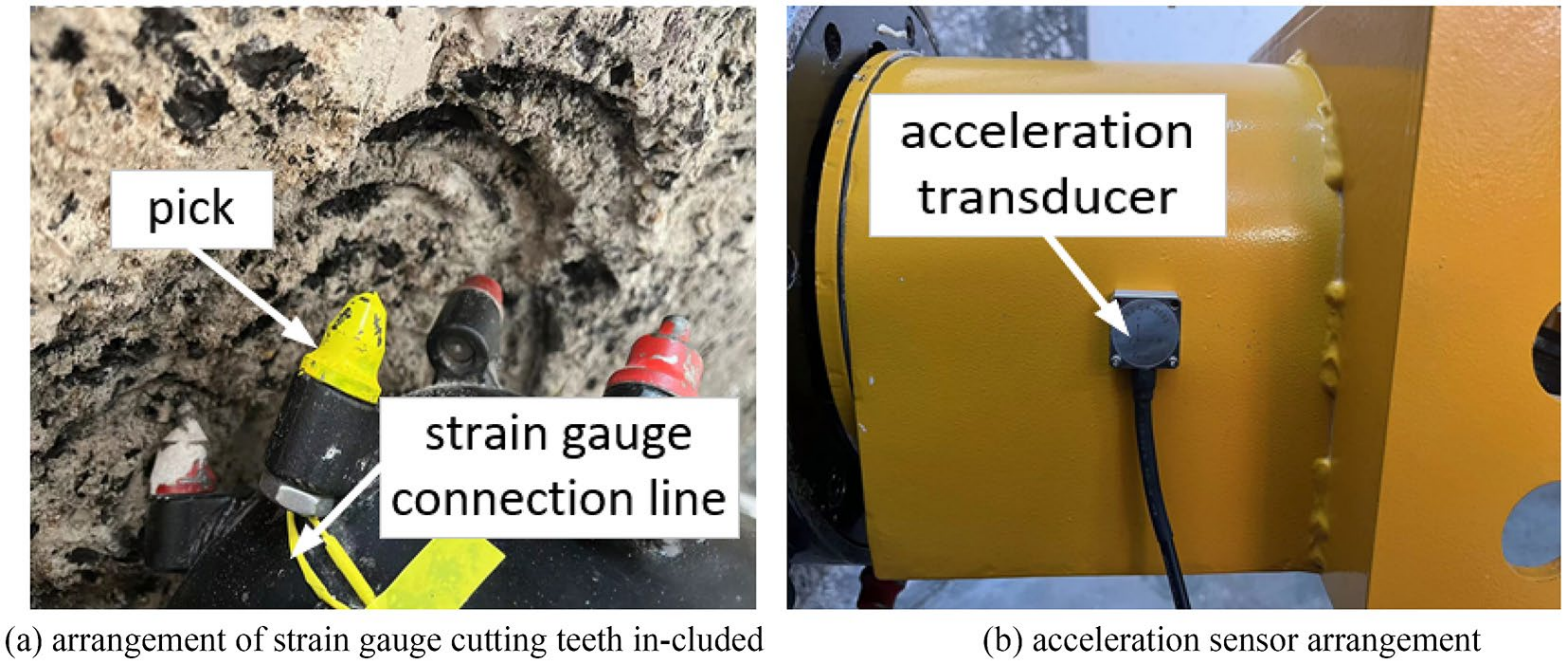
Sensor placement.

In experimental verification, since the vibration of the cutting head is not suitable for direct measurement, the vibration of the front section of the cutting arm is approximately replaced by the vibration of the cutting head during the cutting process. Therefore, in the experiment, the main reliance is on the acceleration sensor to collect the acceleration signal of the cutting head. This article uses the YND-DR-3005 three-dimensional acceleration sensor. Secondly, the test pick selects a pick containing a strain gauge, which can detect the force on the cutting head during the cutting process. The arrangement of the pick and acceleration sensor is shown in Fig. 14.

**Figure 15:**
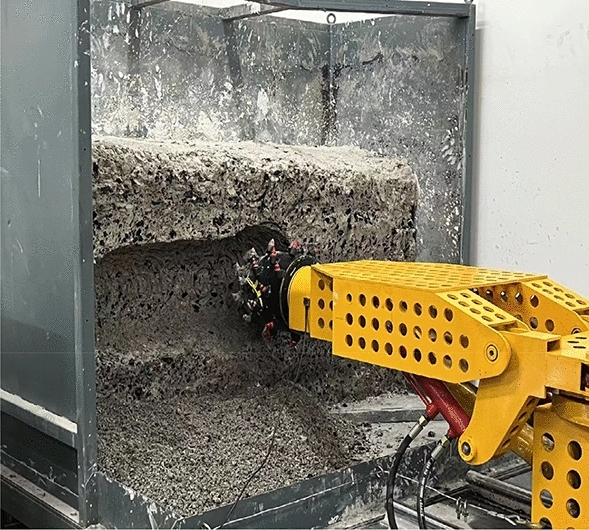
Test cutting process.

**Figure 16:**
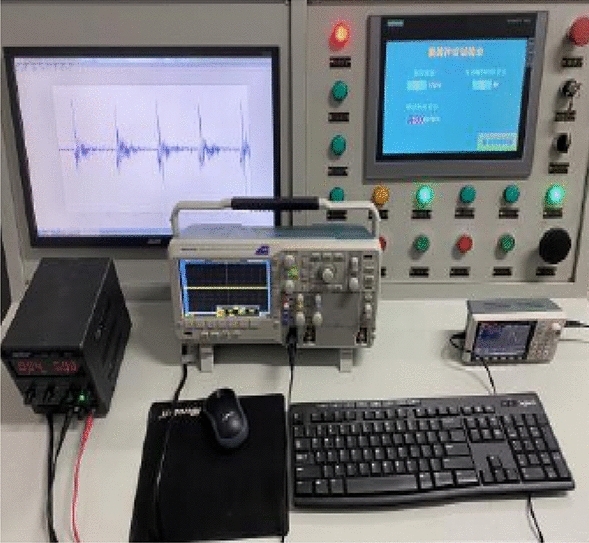
Online test platform.

The experimental cutting process and online testing platform are shown in Figs. 15 and 16. This testing platform not only includes the control cabinet of the mechanical testing system itself, but also includes a switching power supply and an oscilloscope. The switching power supply mainly provides working power for acceleration sensors, strain gauge devices, and signal acquisition circuits. The accelerometer is used to directly read the output signal of the accelerometer online, which is more conducive to improving the sampling frequency of the acceleration signal. The strain collection instrument is used to directly read the signals collected by the internal strain gauge of the pick, facilitating the analysis and processing of subsequent test results. The differential signal output from the acceleration sensor and strain gauge is amplified and converted into a level output through a signal conditioning circuit, and then connected to the oscilloscope through a shielded wire.

**Figure 17:**
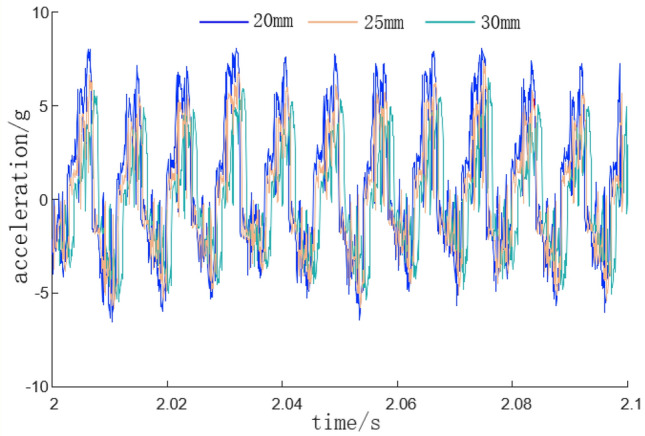
Cutting vibration acceleration curve under different pipeline diameters.

**Figure 18:**
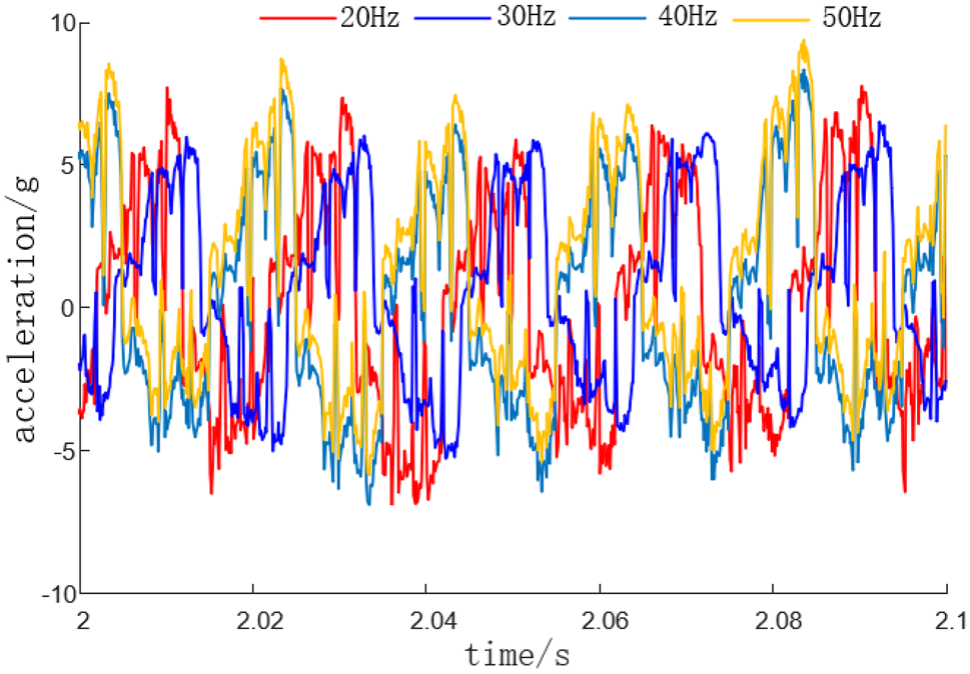
Cutting vibration acceleration curve.

In the cutting test, add active vibration loading. Before the experiment starts, start the cutting motor and hydraulic pump station in advance to ensure the normal rotation of the cutting head and the normal operation of the hydraulic system. Select YND1508 for acceleration signal acquisition and analysis, start DASP acceleration dynamic analysis software, set the system sampling frequency to 8 kHz, the system frequency to 1 Hz, and select continuous recording method for sampling. The cutting process is carried out from left to right, with hydraulic pipeline diameters set to 20 mm, 25 mm, and 30 mm respectively. The vibration frequencies of the hydraulic exciter are 20 Hz, 30 Hz, 40 Hz, and 50 Hz respectively. The measured cutting vibration acceleration curves are shown in Figs. 17 and 18.

According to the acceleration curve of cutting vibration under different pipeline diameters in Fig. 17, it can be seen that, when the hydraulic excitation system is replaced with a pipeline with a diameter of 20 mm, the variation range of cutting head acceleration is − 5.0–6.0 g. When the hydraulic excitation system is replaced with a pipeline with a diameter of 25 mm, the variation range of cutting head acceleration is − 5.0–5.0 g. When the hydraulic excitation system is replaced with a pipeline with a diameter of 30 mm, the variation range of cutting head acceleration is − 5.0–4.0 g. As the inner diameter of the pipeline increases, the range of acceleration change is not large, but the fluctuation at the acceleration turning point decreases in each cycle, which is similar to the simulation results of AMESim software, verifying the authenticity of the simulation results.

From the acceleration curves of cutting vibration at different frequencies in Fig. 18, it can be seen that when a hydraulic exciter is added and the excitation frequency is adjusted to 20 Hz, 30 Hz, and 40 Hz, the cutting vibration system avoids resonance phenomenon. After adding 20 Hz active excitation to the cutting head, the variation range of cutting head acceleration is − 7.0–7.5 g. After adding 30 Hz active excitation to the cutting head, the variation range of cutting head acceleration is − 5.0–6.5 g. After adding 40 Hz active excitation to the cutting head, the variation range of cutting head acceleration is − 7.0–8.7 g. After adding 50 Hz active excitation to the cutting head, the variation range of cutting head acceleration is − 8.5–9.0 g. As the active excitation frequency of the cutting head increases, the acceleration on the cutting head shows a trend of first decreasing and then increasing. At the same time, it can be concluded that when an active excitation frequency of 30 Hz is added, the range of acceleration variation of the cutting head is the smallest, and the inhibitory effect on the acceleration of the cutting head is the strongest.

**Figure 19:**
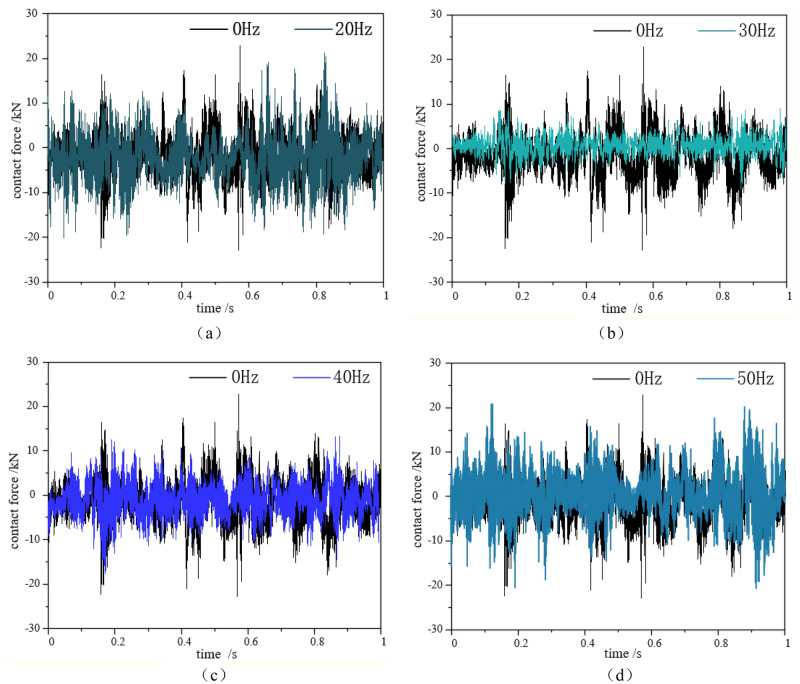
Distribution of Contact Force on Pick under Different Frequencies.

To prove that the frequency of 30 Hz is the optimal auxiliary rock cutting parameter, further contact force tests were conducted on the cutting teeth under different excitation frequencies. The test set the cutting head speed to 36r/min, cutting width to 150 mm, and cutting depth to 50 mm. Measure the cutting force of the cutting teeth during the cutting process using a strain collection instrument, and adjust the output frequencies of the exciter to 0 Hz, 20 Hz, 30 Hz, 40 Hz, and 50 Hz, respectively. The data acquisition and analysis system includes Huasoft YL data acquisition system and DASP dynamic signal analysis system. The contact force of the pick under different excitation frequencies is shown in Fig. 19.

According to the four comparative figures (a), (b), (c), and (d) in Fig. 19, it can be seen that when the excitation system adds a 20 Hz excitation frequency, the contact force on the pick does not change much compared to not adding an excitation frequency. When the excitation system adds a 30 Hz excitation frequency, the range of contact force variation of the pick is small, and the maximum contact force of the pick is 10KN. The cutting process of the cutting system is relatively stable. When the excitation system adds a 40 Hz excitation frequency, the range of contact force variation on the pick increases compared to the range at 30 Hz excitation frequency, and the maximum contact force on the pick is close to 20KN. When the excitation system adds a 50 Hz excitation frequency, the contact force on the pick is almost close to the case without excitation frequency. Therefore, when adding a 30 Hz excitation frequency to the hydraulic excitation system, the contact force on the cutting teeth is minimized, which is more conducive to reducing the damage and wear of the cutting head, and the cutting effect of the cutting head is more obvious.

## Economic benefits

Through research on the usage of EBZ160 cutting heads, it was found that each cutting head consists of 45 cutting teeth, each of which costs approximately 200 yuan. The service life of each cutting tooth does not exceed three months, that is, 4.17 yuan per hour per tooth. After experimental verification, adding active vibration assistance to the cutting system, each cutting tooth can be used for at least four months, that is, 3.13 yuan per hour per tooth. From an economic perspective, The improved hydraulic vibration cutting system has saved 25% compared to before, and there is also a significant improvement in work efficiency.

## Conclusion

This article studies the impact of adding active excitation to the cutting system on vibration characteristics. Firstly, design the structure of the hydraulic exciter and analyze its dynamics and dynamic characteristics. Analyze the impact of key factors on the vibration characteristics of the cutting system through AMESim software. And verify the accuracy of the simulation results and the feasibility of the proposed active vibration assisted rock cutting method through experiments.

1. By analyzing the dynamics of the excitation system, the function relationship between amplitude and pump displacement, frequency, and pipeline inner diameter is obtained.
2. Based on AMESim software, the impact of factors such as pump displacement, excitation frequency, and pipeline parameters on the operational performance of the electro-hydraulic vibration cutting system was analyzed, which is consistent with the theoretical analysis results.
3. Further verify the influence of excitation frequency on the vibration characteristics of the cutting system through experiments. The experiment shows that as the inner diameter of the pipeline increases, although the range of acceleration change is not large, but within each cycle, the fluctuation at the turning point of acceleration decreases. As the excitation frequency increases, the cutting acceleration shows a trend of first decreasing and then increasing, verifying the accuracy of the simulation results.
4. In order to verify that the excitation frequency of 30 Hz is the optimal frequency, further tooth contact force tests were conducted. When adding a 30 Hz excitation frequency to the hydraulic excitation system, the contact force on the cutting teeth is the smallest, which is more conducive to reducing the damage and wear of the cutting head. The cutting effect of the cutting head is more obvious, which is more conducive to rock breaking.
5. Through research on the performance indicators of the cutting teeth used and combined with the actual use of the improved hydraulic excitation system, it is concluded that the service life of the cutting teeth can be extended by about 25% after adding excitation assistance. Therefore, this method has certain economic value.

## Data Availability

All data generated or analyzed in this study are included in this article. Analyze the impact of different factors on the dynamic characteristics of the cutting system using AMESim dynamics software. For more information, please contact the corresponding author.
